# Association between Helicobacter pylori infection and non-alcoholic fatty liver disease for Asian and non-Asian population: A systematic review and meta-analysis

**DOI:** 10.3389/fpubh.2022.1062942

**Published:** 2022-12-08

**Authors:** Zhiyuan Ma, Xiajing Chu, Xiang Yan, Wenjin Wang

**Affiliations:** ^1^Department of Geriatrics Ward 2, The First Hospital of Lanzhou University, Lanzhou, China; ^2^Evidence Based Social Science Research Center, School of Public Health, Lanzhou University, Lanzhou, China; ^3^Health Technology Assessment Center of Lanzhou University, School of Public Health, Lanzhou University, Lanzhou, China; ^4^Evidence-Based Medicine Center, School of Basic Medical Sciences, Lanzhou University, Lanzhou, China; ^5^Key Laboratory of Evidence Based Medicine and Knowledge Translation of Gansu Province, Lanzhou, China; ^6^Department of Emergency Medicine, The First Hospital of Lanzhou University, Lanzhou, China

**Keywords:** Helicobacter pylori infection, non-alcoholic fatty liver disease, risk factors, systematic review, meta-analysis

## Abstract

**Background:**

Several studies have revealed a positive correlation between a Helicobacter pylori (HP) infection and the risk of non-alcoholic fatty liver disease (NAFLD). This meta-analysis was conducted to explore further the relationship between HP infection and NAFLD in the Asian and non-Asian populations.

**Methods:**

Relevant studies published from inception to July 22, 2021, in the following databases: PubMed, EMBASE, the Cochrane library, and Web of Science were comprehensively searched. The odds ratio (OR) and hazard ratio (HR) with a 95% confidence interval (95%CI) were pooled by the random-effects model or fixed-effects model. Additionally, subgroup and sensitivity analyses were performed. The funnel plot and the Egger test were used to estimate publication bias.

**Results:**

This meta-analysis included 25 studies involving 107,306 participants. Positive associations between HP infection and NAFLD were found both for the Asian (OR = 1.30, 95% CI: 1.13–1.49, *P* < 0.01; *I*^2^ = 94.30%, *P* < 0.01) and non-Asian populations (OR = 1.42, 95% CI: 1.04–1.94, *P* = 0.03; *I*^2^ = 44.90%, *P* = 0.09). Moreover, similar results were observed in the Asian female group (OR = 1.31, 95% CI: 1.17–1.46, *P* < 0.01; *I*^2^ = 46.30%, *P* = 0.07) but not for the Asian male group. Subgroup analyses for the Asian population showed that there were differences in the association among NAFLD diagnosis methods (*P* < 0.01) and the study design (*P* < 0.01). However, subgroup and sensitivity analyses results showed that the association for the non-Asian population was not stable enough.

**Conclusions:**

The data obtained in this systematic review and meta-analysis suggested that an HP infection was associated with an increased risk of NAFLD for Asian and non-Asian populations. However, the association was not found for Asian males. Further studies are required to establish the causal association, especially for the non-Asian population.

**Systematic review registration:**

Identifier: CRD42021266871.

## Introduction

Non-alcoholic fatty liver disease (NAFLD) is a chronic liver disease with a prevalence rate of ~25–30%, constantly increasing (1–3). Patients with NAFLD often progress to fibrosis, cirrhosis, hepatocellular carcinoma (HCC), and ultimately death (4). In the coming decades, NAFLD could emerge as the leading cause of mortality due to end-stage liver disease (5). Therefore, NAFLD poses significant healthcare and economic burden to society (5–7).

Several factors are associated with NAFLD. A Helicobacter pylori (HP) infection, which is one of the most frequent gastrointestinal infections may be one of the factors. Approximately 50% of the global population suffers from HP infection (8, 9). In recent years, several studies have tried to elucidate the association between HP and intestinal dysregulation disease (10, 11). Moreover, several epidemiological studies and experimental trials have identified an increased risk of NAFLD among patients infected with HP (3, 12, 13).

Considering the need of strong evidence for risk of NAFLD with HP, meta-analysis and systematic review could be helpful. Meta-analysis is commonly used for achieving high-quality evidence (14–16) and is widely used in natural science and social science research (17, 18). In five recently published meta-analyses, the potential association between HP and NAFLD was explored (19–23). All five meta-analyses showed a positive association between the HP infection and NAFLD risk, but most of the included populations were Asian. Non-Asian populations usually have lower rates of a HP infection, which may prove consistency of this association (24). Subgroup analyses by region for four of these five meta-analyses (19–22) further indicated the positive relationship between HP infection and NAFLD for the Asian population. However, in one of the four meta-analyses, it was found that the relationship was no longer significant in the non-Asian population, and the other three meta-analyses had different results. Several new studies on this topic in the Western population were published (3, 25–28). The results showed that, different from the Asian population, no overall associations were observed between HP and NAFLD (24, 29).

Therefore, this systematic review and meta-analysis were conducted to explore further the relationship between HP infection and NAFLD in the Asian and non-Asian populations to update the evidence and fill in the gaps mentioned above. Besides, we did subgroup analysis based on the different diagnosis methods of NAFLD and HP, the degree of covariate adjustment, the study design, and gender.

## Methods

This systematic review and meta-analysis complied with the Preferred reporting items for systematic reviews and meta-analyses (PRISMA) guidelines (30). This study has been registered in PROSPERO (Registration ID: CRD42021266871).

### Search strategy

The PubMed (1951), EMBASE (1966), the Cochrane Library (2000), and Web of Science (1900) were systematically searched to July 22, 2021 using the medical subject headings (MeSH) and related text words. Supplementary Table 1 presents the detailed search strategy. The reference lists of the original studies included in our analysis were also searched as well as those listed in the published review and meta-analyses (19–23).

### Eligibility criteria and study selection

This study included original publications to evaluate the association between HP infection and the risk of NAFLD. We excluded the followings studies: (1) no information of the country for included population; (2) animal experiments, case reports, case series, reviews, practice guidelines, commentaries, and editorials; (3) unavailable data; (4) non-English or non-Chinese language publications. If more than one publication on the same study population was available, only the most recent publication was included. Two reviewers (Xiajing Chu, Zhiyuan Ma) independently screened the titles and abstracts, selected relevant full texts, and assessed them for eligibility. Non-conformity was resolved by discussion.

### Data extraction

Using a predefined data collection form, two reviewers (Xiajing Chu, Zhiyuan Ma) independently extracted the following data: the first author, year of publication, country of participants, study design, publication type, sample size, participants setting, time of the study, age of participants, methods used for identification, diagnosis of the HP infection and NAFLD, adjusted in the multivariate analysis or non-adjusted effect estimates with the 95% CI. If effect estimates were not provided, the odds ratio (OR) and 95% CI were calculated. Any discrepancies between the reviewers were resolved by discussion, or by resort to a third reviewer (Xiang Yan) if consensus could not be reached.

### Risk of bias assessment

Two independent reviewers (Zhiyuan Ma, Xiang Yan) assessed the risk of bias based on the Newcastle-Ottawa Scale (NOS) (31) for the cohort study and case-control study and the Agency for Healthcare Research and Quality (AHRQ) for the cross-sectional study (32). In the case of disagreement, a third investigator (Xiajing Chu) was consulted. The included cohort studies and case-control studies were rated as “low quality” (0–3 points), “moderate quality” (4–6 points) or “high quality” (7–9 points) based on their overall score on the NOS. The included cross-sectional studies were regarded as high quality (8–11 points), moderate quality (4–6 points), and low quality (< 4 points) based on their overall score on the AHRQ.

### Statistical analyses

This meta-analysis was performed using R software v3.6.1. The odds ratio (OR), hazard ratio (HR) and 95% CI were used to explore the relationship between HP infection and NAFLD. The extent of heterogeneity was interpreted based on the total percentage of variation between the relevant studies, as measured by the *I*^2^ statistical parameter. The heterogeneity was categorized as low if *I*^2^ was 0–25%, moderate if *I*^2^ was 25–50%, and high if *I*^2^ was more than 50% (33). Additionally, Cochrane's *Q*-test was used to assess the presence of heterogeneity. The *P*-value by the Cochrane's *Q*-test > 0.05 indicated no significant heterogeneity among the included studies (22). When the heterogeneity was low, a fixed-effects model was used. Otherwise, a random-effects model was used (19). If necessary, multiple reported analyses per outcome were combined using fixed-effects models, so that each study contributed at most one effect size for each outcome.

Previous studies found a difference in the association of the HP infection with the risk of NAFLD between the Asian and non-Asian populations. Therefore, we analyzed Asian and non-Asian populations, respectively. The Asian population was defined as the population from Asian countries, such as China, India, Iran, Japan, and South Korea. The Non-Asian population was defined as the population from a non-Asian country.

Subgroup and sensitivity analyses were performed to explore the sources of heterogeneity. Subgroup analysis was conducted based on the different diagnosis methods of NAFLD and HP. The common NAFLD methods included liver biopsy, ultrasound, hepatic steatosis index (HIS), NAFLD liver fat score (NAFLD-LFS), and fatty liver index (FLI). HP methods included invasive tests, serology, breath test (UBT), and Stool antigen tests. Furthermore, subgroup analysis was also based on the degree of covariate adjustment, the study design, and gender. Because these factors may have an influence on the overall association between NAFLD and HP based on previous studies (19–23).

Additionally, sensitivity analyses were performed to determine the influence of individual studies on the overall estimates by serially excluding each study.

Funnel plots and the Egger test were used to assess potential publication bias. If the number of included studies were < 10, testing for publication bias was not performed (34).

## Results

### Study selection

**Figure 1:**
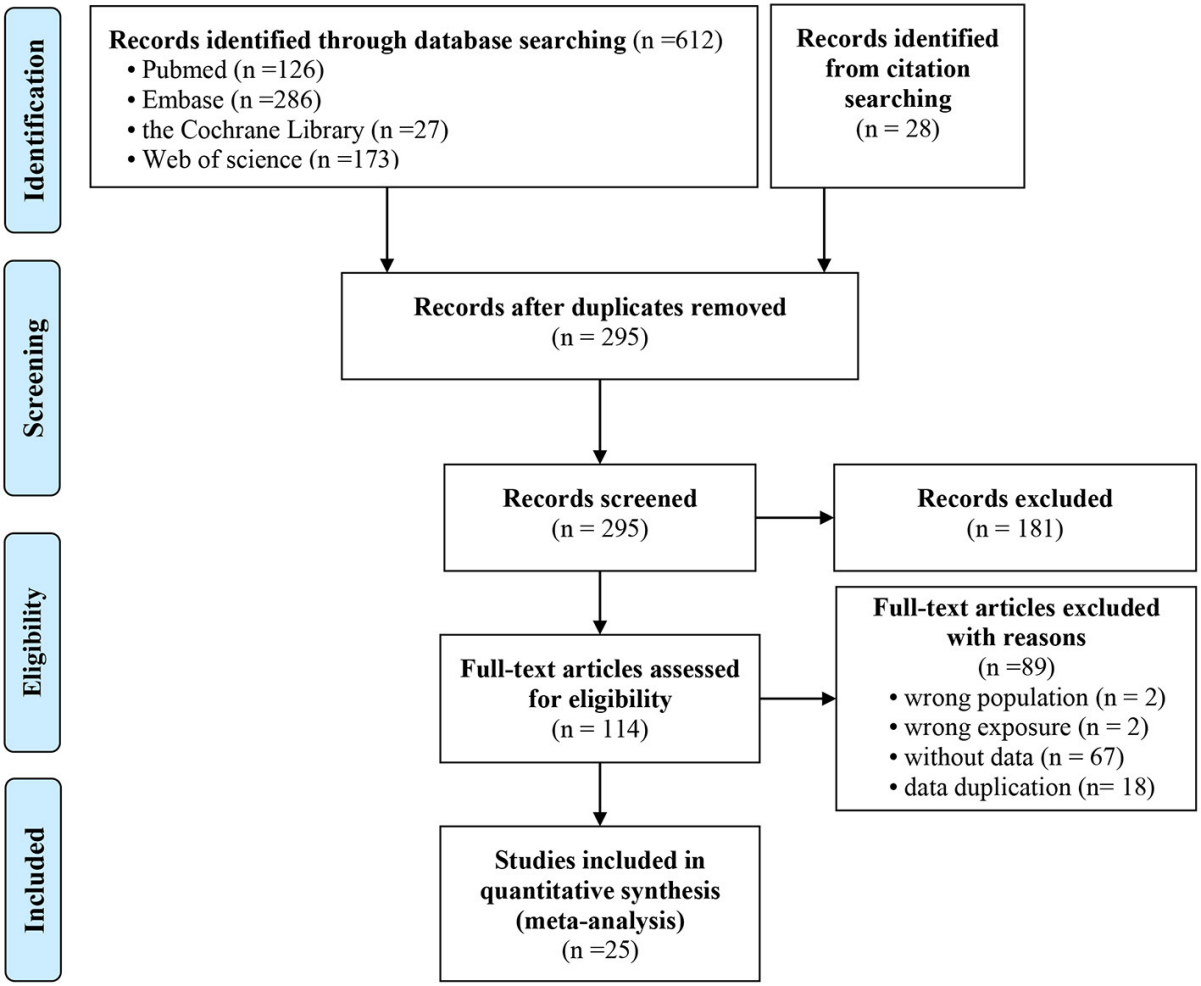
Flowchart presenting the selection process of eligible studies.

For this study, 612 potentially relevant studies were retrieved using the predefined search strategy, and another 28 studies were retrieved through other sources. Among these articles, 345 were duplicate publications. A total of 181 studies were excluded by screening the titles and abstracts. Of the remaining 114 potential eligible articles, 89 studies were excluded by carefully examining the abstracts or full texts. Finally, 25 studies met the inclusion criteria and were included in our meta-analysis. Two studies reported the HR (35, 36), and 23 studies reported the OR (3, 25–29, 37–53). In these 23 studies, one study reported the OR values based on different levels of NAFLD (mild, moderate and severe) (50); another study reported the OR values based on the different white blood cell (WBC) count (52). Therefore, 25 studies were included in this meta-analysis. Figure 1 shows the detailed flowchart of the selection process of eligible studies.

### Study characteristics

Twenty-five studies involving 117,458 participants were included (Table 1). This review contained two cohort studies (35, 36), 17 cross-sectional studies (3, 25–27, 29, 37–39, 41–47, 49, 52), and six case-control studies (28, 40, 48, 50, 51, 53). These studies were published between 2013 and 2021, three of which were conference abstracts (43, 47, 54). The participants from 17 studies were from Asia, and eight studies involved participants who were not from Asia. Regarding the HP test method, two studies used multiple methods. Of the remaining studies, three used only invasive tests, 10 used the breath test, nine used the serology, and one used stool Ag tests. Additionally, most studies (17 studies) used ultrasound to diagnose NAFLD.

**Table 1:** The characteristic of the included studies.

**Study**	**Country of participants**	**Study design**	**Publication type**	**Sample size**	**No. NAFLD**	**Participants setting**	**Study period**	**Age (year) (mean ±SD)**	**Female *n* (%)**	**Mean BMI (kg/m^2^)**	**HP test methods**	**NAFLD diagnosis methods**	**Type of effect size**
Abdel-Razik (35)	Egypt	Cohort	Full article	369	23	University and Hospital	2015.5–2017.12	49.45 ± 8.26	170 (46)	23	SAT	Ultrasound	HR
Baeg et al. (37)	South Korea	Cross-sectional	Full article	3,663	945	Hospital	2010.01–2011.11	53.45 ± 4.06	1,552 (42)	23.6	UBT	HIS or NAFLD-LFS	OR
Cai et al. (38)	China	Cross-sectional	Full article	2,051	433	Hospital	2016.06–2016.12	38.11 ± 10.49	1,337 (65)	23.5	UBT	Ultrasound	OR
Chen et al. (39)	China	Cross-sectional	Full article	2,263	603	Hospital	2013.03–2013.11	69.00 ± 7.00	839 (37)	–	UBT	Ultrasound	OR
Doulberis et al. (40)	Switzerland	Case control	Full article	64	55	Hospital	2017.01–2018.11	47.26 ± 13.04	47 (75)	44.8	Histology	Liver biopsy	OR
Fan et al. (41)	China	Cross-sectional	Full article	21,456	5,213	Hospital	2013.05–2014.06	48.30 ± 15.00	13,782 (67)	23.7	UBT	Ultrasound	OR
Jiang et al. (42)	China	Cross-sectional	Full article	4,081	1,864	–	2005.01–2013.12	44.57 ± 13.56	2,194 (54)	24.5	UBT	Ultrasound	OR
Kang et al. (26)	USA	Cross-sectional	Full article	5,404	1,633	–	2016.11–2017.04	43.00 ± 2.15	2,864 (53)	27	Serology (ELISA)	Ultrasound	OR
Kim et al. (36)	South Korea	Cohort	Full article	17,028	3,381	Medical Center	2016.05–2016.07	49.30 ± 9.30	8,241 (48)	23	Serology (ELISA)	Ultrasound	HR
Kumar et al. (43)	India	Cross-sectional	Abstract	120	27	University	2014	–	–	–	RUT	Ultrasound	OR
Lecube et al. (27)	Spain	Cross-sectional	Full article	416	374	Hospital	2008.06–2010.11	45.50 ± 10.18	304 (73)	44	Histology	Liver biopsy	OR
Lu et al. (44)	China	Cross-sectional	Full article	1,867	596	Hospital	–	54.00 ± 9.60	393 (21)	–	UBT	Ultrasound	OR
Mohammadifard et al. (45)	Iran	Cross-sectional	Full article	130	65	Hospital	2011–2013	37.10 ± 5.85	68 (52)	–	Serology (ELISA)	Ultrasound	OR
Okushin et al. (46)	Japan	Cross-sectional	Full article	5,289	1,802	Medical Center	2014.07–2015.07	48.10 ± 9.02	3,473 (46)	23	Serology (ELISA)	Ultrasound	OR
Polyzos et al. (3)	Greece	Cross-sectional	Full article	53	28	Hospital	2012–2015	54.54 ± 1.83	41 (77)	31	Serology, UBT, history of treatment for H. pylori	Liver biopsy	OR
Shen et al. (47)	China	Cross-sectional	Abstract	9,091	2,371	Hospital	2005.01–2013.12	43.00	4,716 (52)	–	Serology	Ultrasound	OR
Sumida et al. (48)	Japan	Case control	Full article	130	130	Hospital	2011–2013	55.20 ± 14.60	65 (50)	27.5	Serology	Liver biopsy	OR
Tang et al. (28)	USA	Case control	Full article	270	122	Clinic	2014–2015	47.60 ± 12.50	186 (69)	29.9	Histology, serology, or SAT	HIS or NAFLD-LFS	OR
Xu et al. (50)	China	Case control	Full article	17,971	4,825	Hospital	2020.07–2014.06	45 ± 18	5,898 (32.8)	24.0	Serology (ELISA)	Ultrasound	OR
Yu et al. (52)	China	Cross-sectional	Full article	20,389	7,592	Hospital	2015.01–2015.12	47.85 ± 13.20	8,420 (41)	23.7	UBT	Ultrasound	OR
Zhang et al. (53)	China	Case control	Full article	1,200	600	Hospital	2010.06–2014.07	–	–	–	UBT	Liver biopsy	OR
Alvarez et al. (29)	Guatemala	Cross-sectional	Full article	424	264	Community	2016.04–2016.10	55.30 ± 3.75	253 (59.7)	–	Serology	HIS or FLI	OR
Abo-Amer et al. (25)	Egypt	Cross-sectional	Full article	646	524	Hospital	2019.06–2019.10	36.65 ± 11.15	319 (49)	29.2	Serology (ELISA)	Ultrasound	OR
Wang et al. (49)	China	Cross-sectional	Full article	1,898	505	Hospital	2018.07–2018.10	37.19 ± 0.17	681 (40)	23.1	UBT	Ultrasound	OR
Yan et al. (51)	China	Case control	Full article	1,185	529	Hospital	2017.01–2019.06	42.06 ± 10.78	407 (34)	24.7	UBT	Ultrasound	OR

ELISA, enzyme-linked immunosorbent assay; RUT, rapid urease test; HIS, hepatic steatosis index; NAFLD-LFS, NAFLD liver fat score; FLI, the fatty liver index; UBT, Breath test; SAT, Stool antigen tests; HR, Hazard ratio; OR, Odds ratio.

### Quality assessment

In this study, quality assessments were conducted on full texts. Supplementary Table 2 presents the quality assessment results according to NOS for the cohort study and the case-control study. The scores of included studies ranged from six to nine (mean 7.75), and the comparability had a low score for most included studies. Supplementary Table 3 presents the results of AHRQ for the cross-sectional study. The scores of included studies ranged from one to 15 (mean 6.53). In only one study, the handling of missing data in the analysis was explained (49), and in only one study, the patient response rates and completeness of the data collector were summarized (41).

### HP infection and NAFLD for Asian and non-Asian

**Figure 2:**
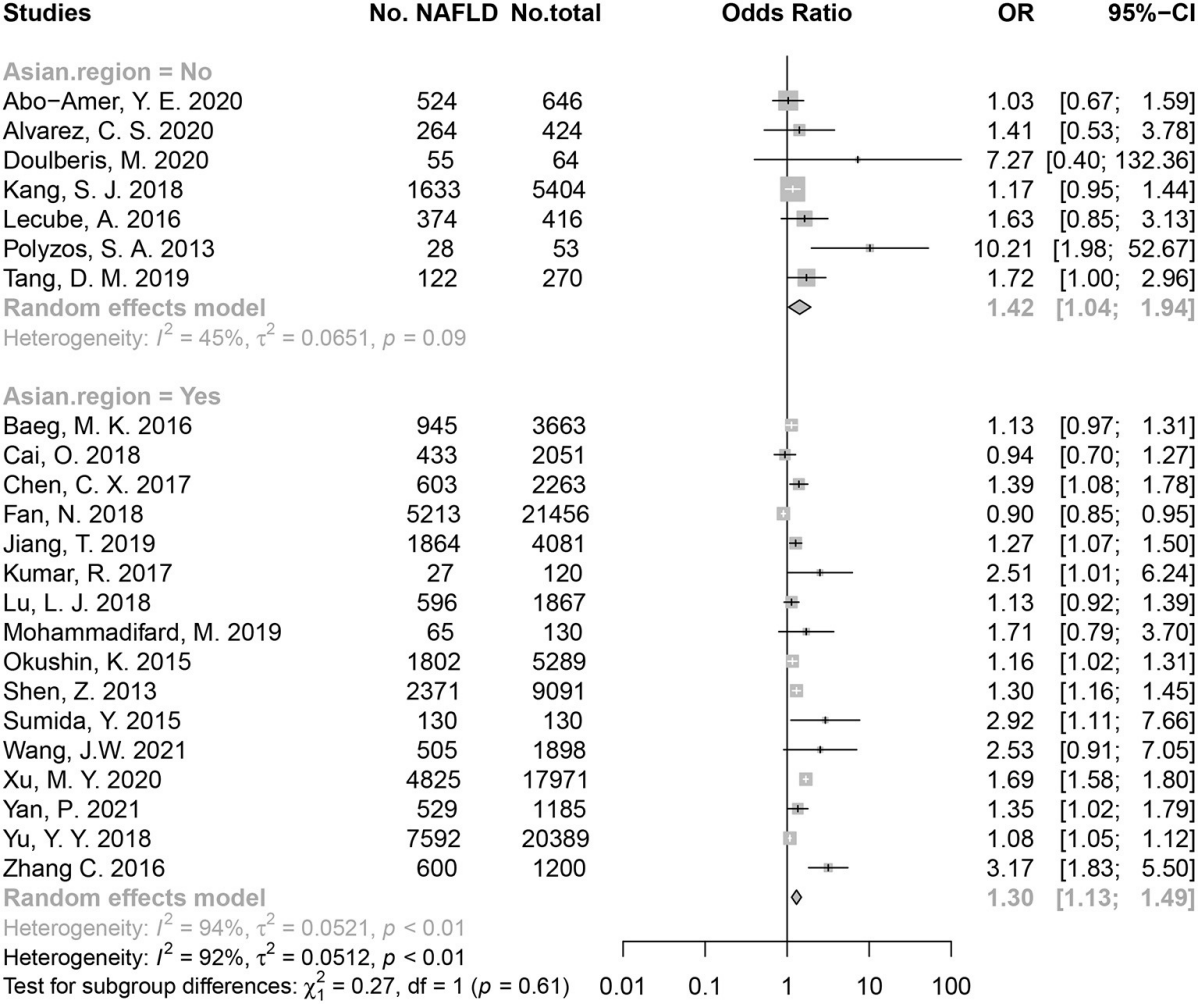
The associations between HP infection and NAFLD for Asian and non-Asian populations.

In an overall pooled analysis, HP-positive populations had a higher risk of NAFLD than HP-negative populations (OR = 1.30) both in Asian and non-Asian countries. Regarding The Asian population, the meta-analysis using random-effects model showed HP infection was associated with a risk of 1.30 of developing NAFLD (OR = 1.30, 95% CI: 1.13–1.49, *P* < 0.01; *I*^2^ = 94.30%, *P* < 0.01; Figure 2). The HP infection was also associated with an increase risk of NAFLD in non-Asian population (OR = 1.42, 95% CI: 1.04–1.94, *P* = 0.03; *I*^2^ = 44.90%, *P* = 0.09; Figure 2). No significant difference in the association was found between non-Asia and Asia populations (*P* = 0.61). Besides, two studies reported the effect size of HR (35, 36), and the pooled HR supported the association between HP infection and risk of NAFLD (HR = 1.13, 95% CI: 1.04–1.23) (Supplementary Figure 1).

### Subgroup analysis for the Asian population

#### Country of population

Table 2 shows the subgroup analyses of the Asian population for 16 studies that used OR as an effect size. The HP infection was associated with the risk of NAFLD (OR = 1.29, 95% CI: 1.09–1.51, *P* < 0.01; *I*^2^ = 96.10%, *P* < 0.01) for Chinese (11 studies, 36,942 participants). Subgroup analysis showed no significant difference among countries (*P* = 0.29).

**Table 2:** Subgroup analyses for association between HP infection and NAFLD for Asian.

**Subgroups**	**No. studies**	**Sample size**	**Effect size**		**Heterogeneity**		***P*-interaction**
			**OR (95%CI)**	***P*-value**	***I^2^* (%)**	***P*-value**	
**Country of population**							0.29
China	11	83,452	1.29 (1.09, 1.51)	< 0.01	96.10	<0.01	
India	1	120	2.51 (1.01, 6.24)	0.05	–	–	
Iran	1	130	1.71 (0.79, 3.70)	0.17	–	–	
Japan	2	5,419	1.62 (0.68, 3.86)	0.28	71.10	0.06	
South Korea	1	3,663	1.13 (0.97, 1.31)	0.11	–	–	
**HP test methods**							0.08
Invasive Tests	1	120	2.51 (1.01, 6.24)	0.05	–	–	
Serology	5	32,611	1.44 (1.15, 1.80)	<0.01	89.30	<0.01	
UBT	10	60,053	1.17 (1.04, 1.32)	<0.01	86.90	<0.01	
**NAFLD diagnosis methods**							<0.01
Liver biopsy	2	1,330	3.11 (1.93, 5.01)	<0.01	0.00	0.88	
Multiple methods	1	3,663	1.13 (0.97, 1.31)	0.11	–	–	
Ultrasound	13	87,791	1.25 (1.08, 1.44)	<0.01	95.10	<0.01	
**Degree of covariate adjustment**						0.21
No	6	36,886	1.18 (1.06, 1.31)	<0.01	66.60	0.01	
Yes	10	55,898	1.41 (1.09, 1.82)	<0.01	96.30	<0.01	
**Study design**							<0.01
Case-control study	4	20,486	1.82 (1.37, 2.42)	<0.01	65.50	0.03	
Cross sectional study	12	72,298	1.15 (1.04, 1.27)	<0.01	84.60	<0.01	
**Gender**							0.11
Female	8	38,507	1.31 (1.17, 1.46)	<0.01	46.30	0.07	
Male	8	38,507	1.00 (0.73, 1.36)	0.98	95.10	<0.01	

#### HP test methods

For the three main HP test methods, the positive association between HP infection and the risk of NAFLD were observed in one study that used invasive tests (OR = 2.51, 95% CI 1.01–6.24, *P* = 0.05; Table 2), in five studies that used serology (OR = 1.44, 95% CI 1.15–1.80, *P* < 0.01; *I*^2^ = 89.30%, *P* < 0.01; Table 2), and in ten studies that used UBT (OR = 1.17, 95% CI 1.04–1.32, *P* < 0.01; *I*^2^ = 86.90%, *P* < 0.01; Table 2). Subgroup analysis showed significant differences among invasive tests, serology, and UBT groups (*P* = 0.08; Table 2).

#### NAFLD diagnosis methods

For the three main NAFLD diagnosis methods, the positive association between HP infection and the risk of NAFLD were observed in two studies that used liver biopsy (OR = 3.11, 95% CI 1.93–5.01, *P* < 0.01; *I*^2^ = 0.00%, *P* = 0.88; Table 2), and in 13 studies that used ultrasound (OR = 1.25, 95% CI 1.08–1.44, *P* < 0.01; *I*^2^ = 95.10%, *P* < 0.01; Table 2). Subgroup analysis showed a significant difference among liver biopsy, multiple methods, and ultrasound groups (*P* < 0.01; Table 2).

#### Degree of covariate adjustment

The meta-analysis on six studies with 33,770 participants without covariate adjustment showed the positive association between HP infection and the risk of NAFLD (OR = 1.18, 95% CI 1.06–1.31, *P* < 0.01; *I*^2^ = 66.60%, *P* = 0.01; Table 2). The positive association between HP infection and the risk of NAFLD was confirmed by 10 studies with 34,213 participants with covariate adjustment (OR = 1.41, 95% CI 1.09–1.82, *P* < 0.01; *I*^2^ = 96.30%, *P* < 0.01; Table 2), the adjusted covariates included sex, age, education level, medical history, lifestyles, and biomarks (see Supplementary Table 4). Subgroup analysis showed no significant differences between studies without covariate adjustment and with covariate adjustment groups (*P* = 0.21; Table 2).

#### Study design

Subgroup analysis showed a significant difference between case-control studies and cross-sectional studies (*P* < 0.01; Table 2), although neither group found the positive association between HP infection and the risk of NAFLD. The pooled OR of four case-control studies with 7,480 participants was 1.82 (OR = 1.82, 95% CI 1.37–2.42, *P* < 0.01; *I*^2^ = 65.50%, *P* = 0.03; Table 2). The pooled OR from 12 studies with 60,503 participants (cross-sectional studies) was 1.15 (OR = 1.15, 95% CI 1.04–1.27, *P* < 0.01; *I*^2^ = 84.60%, *P* < 0.01; Table 2).

#### Gender

Subgroup analysis based on gender showed the HP infection had positive association with risk of NAFLD in female group (OR = 1.31, 95% CI: 1.17–1.46, *P* < 0.01; *I*^2^ = 46.30%, *P* = 0.07) but not in male group (*P* = 0.98). Subgroup analysis showed no significant differences between males and females (*P* = 0.11; Table 2).

### Subgroup analysis in non-Asian countries

#### Country of population

Table 3 shows the subgroup analyses of the non-Asian population for seven studies that used OR as an effect size. Subgroup analysis showed no significant differences among countries (*P* = 0.10; Table 3). No association between the HP infection and NAFLD risk was found except for Greece (Table 3).

**Table 3:** Subgroup analyses for association between HP infection and NAFLD for non-Asian.

**Subgroups**	**No. studies**	**Sample size**	**Effect size**		**Heterogeneity**		***P*-interaction**
			**OR (95%CI)**	***P*-value**	***I*^2^ (%)**	***P*-value**	
**Country of population**							0.10
Egypt	1	646	1.03 (0.67, 1.59)	0.89	–	–	
Greece	1	53	10.21 (1.98, 52.67)	<0.01	–	–	
Guatemala	1	424	1.41 (0.53, 3.78)	0.49	–	–	
Spain	1	416	1.63 (0.85, 3.13)	0.14	–	–	
Switzerland	1	64	7.27 (0.40, 132.36)	0.18	–	–	
US	2	5,674	1.30 (0.93, 1.83)	0.13	41.10	0.19	
**HP test methods**							0.22
Invasive Tests	2	480	1.75 (0.93, 3.31)	0.08	0.00	0.32	
Multiple methods	2	323	3,52 (0.64, 19.47)	0.15	75.50	0.04	
Serology	3	6,474	1.15 (0.96, 1.38)	0.13	0.00	0.80	
**NAFLD diagnosis methods**							0.11
Liver biopsy	3	533	3.76 (0.93, 15.15)	0.06	58.50	0.09	
Multiple methods	2	694	1.64 (1.02, 2.64)	0.04	0.00	0.73	
Ultrasound	2	6,050	1.14 (0.95, 1.38)	0.16	0.00	0.60	
**Degree of covariate adjustment**						0.25
No	4	1,176	2.02 (0.88, 4.63)	0.10	66.50	0.03	
Yes	3	6,098	1.23 (1.02, 1.49)	0.03	0.00	0.41	
Study design							0.36
Case-control study	2	334	1.81 (1.06, 3.08)	0.03	0.00	0.34	
Cross sectional study	5	6,942	1.34 (0.94, 1.91)	0.11	50.10	0.09	

#### HP test methods

For the three main HP test methods, no association between HP infection and the risk of NAFLD were observed in two studies that used invasive tests (OR = 1.75, 95% CI 0.93–3.31, *P* = 0.08; *I*^2^ = 0.00%, *P* = 0.32; Table 3), in three studies that used serology (OR = 3.52, 95% CI 0.64–19.47, *P* = 0.15; *I*^2^ = 75.50%, *P* = 0.04; Table 3), or in two studies that used multiple methods (OR = 1.15, 95% CI 0.96–1.38, *P* = 0.13; *I*^2^ = 0.00%, *P* = 0.80; Table 3). Subgroup analysis showed no significant differences among invasive tests, serology and multiple methods groups (*P* = 0.22; Table 3).

#### NAFLD diagnosis methods

For the three main NAFLD diagnosis methods, the positive association between HP infection and the risk of NAFLD were observed in two studies that used multiple methods (OR = 1.64, 95% CI 1.02–2.64, *P* = 0.04; *I*^2^ = 58.50%, *P* = 0.09; Table 3) but not for the studies using serology (*P* = 0.13) and multiple methods (*P* = 0.15). Subgroup analysis showed no significant differences among liver biopsy, multiple methods and ultrasound groups (*P* = 0.11; Table 3).

#### Degree of covariate adjustment

The meta-analysis on four studies with 33,770 participants without covariate adjustment showed no association between the HP infection and the risk of NAFLD (OR = 2.02, 95% CI 0.88–4.63, *P* = 0.10; *I*^2^ = 66.50%, *P* = 0.03; Table 3). But positive association between HP infection and the risk of NAFLD was observed for three studies with 34,213 participants with covariate adjustment (OR = 1.23, 95% CI 1.02–1.49, *P* = 0.03; *I*^2^ = 0.00%, *P* = 0.41; Table 3), the adjusted covariates included sex, age, education level, medical history, lifestyles, and biomarks (see Supplementary Table 4). The subgroup analysis showed no significant difference was observed among studies without covariate adjustment and with covariate adjustment groups (*P* = 0.25; Table 3).

#### Study design

Subgroup analysis showed no significant differences among case-control studies and cross-sectional studies groups (*P* = 0.36; Table 3). The positive association between HP infection and the risk of NAFLD was observed for two case-control studies with 31,834 participants (OR = 1.81, 95% CI 1.06–3.08, *P* = 0.03; *I*^2^ = 0.00%, *P* = 0.34; Table 3). But no association was observed for five cross-sectional studies with 5,448 participants (OR = 1.34, 95% CI 0.94–1.91, *P* = 0.11; *I*^2^ = 50.10%, *P* = 0.09; Table 3).

### Sensitivity analysis and publication bias

Sensitivity analysis was performed by removing one study at a time, and the *P*-value confirmed the stability of the results for the Asian population. However, the results of the non-Asian population were not stable enough (Supplementary Figures 1–3). Analysis of the funnel plot of the OR for publication bias suggested the absence of bias because of plot symmetry (Supplementary Figure 4). Furthermore, the Egger test showed no publication bias (*P* = 0.17).

## Discussion

### Main findings

This meta-analysis included 25 studies (two control studies, six case-control studies, and 17 cross-sectional studies) involving 107,306 participants from 11 countries. The data studies showed a positive association between HP and the risk of development of NAFLD both for Asian and non-Asian populations. Similar results were observed in most subgroups of the Asian populations, except for the male group. However, the association for the non-Asian population was not stable enough.

### Potential explanations and implications

In several studies, the underlying mechanism involved in HP infection and the risk of NAFLD development were explained. HP infection is known to cause chronic low-grade systemic inflammation by increasing the levels of proinflammatory cytokines (55). Additionally, HP influences the development of NAFLD through hormonal effects (13). Moreover, HP-induced bacterial translocation in chronic liver disease can be detected by human β-defensin-1(40).

A prospective multicenter pilot cohort study (35) showed that after eradication therapy of HP infection, there was a significant reduction in levels of C-reactive protein, leptin, insulin resistance, NAFLD-LFS, TNF-α, and IL-6. After a 24-month follow-up, the incidence rate of NAFLD in patients with eradication therapy was five times lower compared to that in untreated patients. Additionally, a randomized controlled trial (56) reported a significant improvement in insulin resistance after 24 weeks of successful eradication of HP. Thus, the eradication of HP had an advantageous effect on metabolic diseases, such as NAFLD. These findings were consistent with our results. Although the forest plot of the OR both for Asian and non-Asian populations showed a moderate or high heterogeneity, the sensitivity analysis and publication bias substantiated the robustness of our results for the Asian population. NAFLD diagnosis methods and study design contributed to the heterogeneity of the association for the Asian population.

Subgroup analysis based on different country populations showed positive associations between HP infection and the risk of NAFLD in China, India, and Greece. Considering the small size effect, we thought that the positive association in China was more robust. This observation was consistent with the data presented in previous studies (24, 29). The pathogenesis of NAFLD is known to include adipose tissue-derived hormones, nutritional factors, genetic and epigenetic factors, gut microbiota, and insulin resistance (57, 58). Because some information on these pathogenesis factors and metabolic risk factors (e.g., body weight, hypertension, hyperglycemia, and dyslipidemia) was lacking, subgroup analysis based on these factors was not conducted. Further studies are required to verify these results by well-designed human studies, considering the complex interactions with confounding factors, such as environmental and genetic susceptibility factors (19). Additionally, we found that females with HP infection had a higher risk of NAFLD in the Asian population. Although the underlying mechanism of action is unclear, many studies have shown that gender is a factor influencing the risk of an infection (36, 41, 48). Besides, the observed differences in associations are most likely due to social determinants of health, but not decided by biological or genetic factor only. The difference on associations between Asian and non-Asian population were possibly caused by adoption of dietary and lifestyle habits, crowded living conditions, poor sanitation, and lack of access to care (24, 59). However, the small sample size in our included populations might be a confounding variable.

### Comparison with previous work

Five studies (19–23) assessed the association between HP infection and NAFLD, and all studies concluded that the presence of a significant relationship between HP and NAFLD, and one study noted a 36% increased risk of NAFLD in patients with HP infection (20). HP infection indeed showed a positive association with NAFLD for the Asian population. These findings were similar to the findings of our study. Compared with previous meta-analyses, this study used a larger sample size to explore the association between HP infection and NAFLD in the non-Asian population than in a previous Meta-analysis. In our review, we included additional studies, including cross-sectional, case-control, and cohort studies. The comprehensive subgroup analysis, which was based on the region of the population, the different diagnosis methods of NAFLD and HP, the degree of covariate adjustment, and the study design, confirmed the robustness of our results.

### Strengths and limitations

A specific subgroup analysis was performed based on the country of the population, the different diagnosis methods used for NAFLD and HP, the degree of covariate adjustment, and the study design. These methods supported our conclusions. Our study had several limitations. First, the included studies had a small sample size of fewer than 500 participants, which might affect the quality of evidence. Second, the bias of the included retrospective studies might affect the quality of evidence. Additionally, the Asian and Non-Asian populations were defined as a population from the Asian or Non-Asian countries, respectively. The designation may not be reliable enough, but there is no more precise approach due to lack of data. Finally, subgroup analyses were performed based on potential confounding variables, but subgroup analyses by several important pathogenesis factors, HP test ranges, and metabolic risk factors (e.g., body weight, hypertension, hyperglycemia, and dyslipidemia) could not be conducted because of lacking information. Further prospective studies are required to perform an in-depth analysis of the heterogeneity.

## Conclusions

This systematic review and meta-analysis suggested that HP infection was associated with an increased risk of NAFLD in Asian and non-Asian populations. However, the association was not found for Asian males; the association for the non-Asian population was not stable enough. Further studies are required to establish a causal association between HP infection and NAFLD. Thus, eradicating HP infection might be a new approach to the clinical prevention and treatment of NAFLD.

## Data availability statement

The data analyzed in this study is subject to the following licenses/restrictions: The datasets used and/or analyzed during the current study are available from the corresponding author on reasonable request. Requests to access these datasets should be directed to wwjou@126.com.

## Author contributions

ZM and XC conceptualized and designed the protocol, drafted the initial manuscript, and reviewed the manuscript. XY and ZM defined the concepts and search items and data extraction process as well as methodological appraisal of the studies. XC, XY, and WW planned the data extraction and statistical analysis. XY and WW provided critical insights. All authors have approved and contributed to the final written manuscript.
