# Supplementary material for: Association between Helicobacter pylori infection and non-alcoholic fatty liver disease for Asian and non-Asian population: A systematic review and meta-analysis
Source: Front Public Health. 2022 Dec 8;10:1062942. doi: 10.3389/fpubh.2022.1062942 (PMC9773836; doi:10.3389/fpubh.2022.1062942)

**Table S1 The overall search strategy**

**Table S2 NOS Quality assessment of included cohort studies and cross-sectional studies**

**Table S3 AHQE of included case control studies**

**Table S4 The covariates in included studies**

**Figure S1 The sensitivity analysis of the meta-analysis in association between HP infection and NAFLD with HR**

**Figure S2 The sensitivity analysis of the meta-analysis in association between HP infection and NAFLD with OR for Asian**

**Figure S3 The sensitivity analysis of the meta-analysis in association between HP infection and NAFLD with OR for non-Asian**

**Figure S4 The publication bias**

**Table S1**

| **Databases** | **Search strategies** |
| --- | --- |
| **Pubmed** | **#1** "Helicobacter pylori"[Mesh]  **#2** ((((("Campylobacter pylori*"[Title/Abstract]) OR ("Helicobacter pylori*"[Title/Abstract])) OR ("Helicobacter pylori infection*"[Title/Abstract])) OR ("Campylobacter pylori infection*"[Title/Abstract])) OR ("H. pylori"[Title/Abstract])) OR ("HP"[Title/Abstract])  **#3** "Non-alcoholic Fatty Liver Disease"[Mesh]  **#4** ((((((((((((((("Non alcoholic Fatty Liver*"[Title/Abstract]) OR ("Nonalcoholic Fatty Liver*"[Title/Abstract])) OR ("Nonalcoholic Steatohepatiti*"[Title/Abstract])) OR ("Non alcoholic Steatohepatiti*"[Title/Abstract])) OR ("Nonalcoholic liver disease*"[Title/Abstract])) OR ("Non alcoholic liver disease*"[Title/Abstract])) OR ("Non alcoholic hepatosteatosis"[Title/Abstract])) OR ("Nonalcoholic hepatosteatosis"[Title/Abstract])) OR ("Non alcoholic liver steatosis"[Title/Abstract])) OR ("Nonalcoholic liver steatosis"[Title/Abstract])) OR ("Non alcoholic hepatic steatosis"[Title/Abstract])) OR ("Nonalcoholic hepatic steatosis"[Title/Abstract])) OR ("Nonalcoholic FLD"[Title/Abstract])) OR ("NAFL"[Title/Abstract])) OR ("NASH"[Title/Abstract])) OR ("NAFLD"[Title/Abstract])  **#5** #1 OR #2  **#6** #3 OR #4  **#7** #5 AND #6 |
| **EMBASE** | **#1** 'helicobacter pylori'/exp  **#2** 'helicobacter pylori':ti,ab,kw OR 'campylobacter pylori*':ti,ab,kw OR 'helicobacter pylori*':ti,ab,kw OR 'helicobacter pylori infection*':ti,ab,kw OR 'campylobacter pylori infection*':ti,ab,kw OR 'h. pylori':ti,ab,kw OR 'hp':ti,ab,kw  **#3** 'nonalcoholic fatty liver'/exp  **#4** 'non alcoholic fatty liver*':ti,ab,kw OR 'nonalcoholic fatty liver*':ti,ab,kw OR 'nonalcoholic steatohepatiti*':ti,ab,kw OR 'non alcoholic steatohepatiti*':ti,ab,kw OR 'nonalcoholic liver disease*':ti,ab,kw OR 'non alcoholic liver disease*':ti,ab,kw OR 'non alcoholic hepatosteatosis':ti,ab,kw OR 'nonalcoholic hepatosteatosis':ti,ab,kw OR 'non alcoholic liver steatosis':ti,ab,kw OR 'nonalcoholic liver steatosis':ti,ab,kw OR 'non alcoholic hepatic steatosis':ti,ab,kw OR 'nonalcoholic hepatic steatosis':ti,ab,kw OR 'nonalcoholic fld':ti,ab,kw OR 'non alcoholic fld':ti,ab,kw OR 'nafl':ti,ab,kw OR 'nash':ti,ab,kw OR 'nafld':ti,ab,kw OR 'nonalcoholic fatty liver':ti,ab,kw  **#5** #1 OR #2  **#6** #3 OR #4  **#7** #5 AND #6 |
| **The Cochrane Library** | **#1** MeSH descriptor: [Helicobacter pylori] explode all trees  **#2** ("Helicobacter pylori"):ti,ab,kw OR ("Campylobacter pylori*"):ti,ab,kw OR ("Helicobacter pylori*"):ti,ab,kw OR ("Helicobacter pylori infection*"):ti,ab,kw OR ("Campylobacter pylori infection*"):ti,ab,kw (Word variations have been searched)  **#3** ("H. pylori"):ti,ab,kw OR ("HP"):ti,ab,kw (Word variations have been searched)  **#4** #1 OR #2 OR #3  **#5** MeSH descriptor: [Non-alcoholic Fatty Liver Disease] explode all trees  **#6** ("Non alcoholic Fatty Liver*"):ti,ab,kw OR ("Nonalcoholic Fatty Liver*"):ti,ab,kw OR ("Nonalcoholic Steatohepatiti*"):ti,ab,kw OR ("Non alcoholic Steatohepatiti*"):ti,ab,kw OR ("Nonalcoholic liver disease*"):ti,ab,kw (Word variations have been searched)  **#7** ("Non alcoholic liver disease*"):ti,ab,kw OR ("Non alcoholic hepatosteatosis"):ti,ab,kw OR ("Nonalcoholic hepatosteatosis"):ti,ab,kw OR ("Non alcoholic liver steatosis"):ti,ab,kw OR ("Nonalcoholic liver steatosis"):ti,ab,kw (Word variations have been searched)  **#8** ("Non alcoholic hepatic steatosis"):ti,ab,kw OR ("Nonalcoholic hepatic steatosis"):ti,ab,kw OR ("NAFL"):ti,ab,kw OR ("Nonalcoholic FLD"):ti,ab,kw OR ("Non alcoholic FLD"):ti,ab,kw (Word variations have been searched)  **#9** ("NASH"):ti,ab,kw OR ("NAFLD"):ti,ab,kw (Word variations have been searched)  **#10** #5 OR #6 OR #7 OR #8 OR #9  **#11** #4 AND #10 |
| **Web of Science** | **#1** TS=("Helicobacter pylori") OR TS=("Campylobacter pylori*") OR TS=("Helicobacter pylori*") OR TS=("Helicobacter pylori infection*") OR TS=("Campylobacter pylori infection*") OR TS=("H. pylori") OR TS=("HP")  **#2** TS=("Non-alcoholic Fatty Liver Disease") OR TS=("Non alcoholic Fatty Liver*") OR TS=("Nonalcoholic Fatty Liver*") OR TS=("Nonalcoholic Steatohepatiti*") OR TS=("Non alcoholic Steatohepatiti*") OR TS=("Nonalcoholic liver disease*") OR TS=("Non alcoholic liver disease*") OR TS=("Non alcoholic hepatosteatosis") OR TS=("Nonalcoholic hepatosteatosis") OR TS=("Non alcoholic liver steatosis") OR TS=("Nonalcoholic liver steatosis") OR TS=("Non alcoholic hepatic steatosis") OR TS=("Nonalcoholic hepatic steatosis") OR TS=("Nonalcoholic FLD") OR TS=("Non alcoholic FLD") OR TS=("NAFL") OR TS=("NASH") OR TS=("NAFLD")  **#3** #1 AND #2 |

**Table S2 NOS Quality assessment of included cohort studies and case control studies**

| **Studies** | **Selection** | **Comparability** | **Exposure/Outcome** | **NOS scores** |
| --- | --- | --- | --- | --- |
| Abdel-Razik, A. 2018 | 4 | 2 | 3 | 9 |
| Doulberis, M. 2020 | 4 | 2 | 3 | 9 |
| Kim, T. J. 2017 | 3 | 1 | 2 | 6 |
| Sumida, Y. 2015 | 3 | 2 | 2 | 7 |
| Tang, D. M. 2019 | 3 | 2 | 3 | 8 |
| Xu, M. Y. 2020 | 3 | 2 | 3 | 8 |
| Zhang C. 2016 | 4 | 2 | 1 | 8 |
| Yan, P. 2021 | 3 | 2 | 2 | 7 |

**Table S3** **AHQE of included cross-sectional studies**

| **Studies** | **A** | **B** | **C** | **D** | **E** | **F** | **G** | **H** | **I** | **J** | **K** | **Total** |
| --- | --- | --- | --- | --- | --- | --- | --- | --- | --- | --- | --- | --- |
| Baeg, M. K. 2016 | 1 | 1 | 1 | 0 | 0 | 1 | 1 | 1 | 0 | 0 | 1 | 7 |
| Cai, O. 2018 | 1 | 1 | 1 | 0 | 0 | 1 | 1 | 1 | 0 | 0 | 1 | 7 |
| Chen, C. X. 2017 | 1 | 1 | 1 | 0 | 0 | 1 | 0 | 1 | 0 | 0 | 1 | 6 |
| Fan, N. 2018 | 1 | 1 | 1 | 0 | 0 | 1 | 1 | 1 | 0 | 1 | 1 | 9 |
| Jiang, T. 2019 | 0 | 1 | 0 | 0 | 0 | 1 | 0 | 1 | 0 | 0 | 0 | 3 |
| Kang, S. J. 2018 | 1 | 1 | 1 | 0 | 0 | 1 | 1 | 1 | 0 | 0 | 0 | 6 |
| Lecube, A. 2016 | 1 | 1 | 1 | 0 | 0 | 1 | 1 | 1 | 0 | 0 | 0 | 6 |
| Lu, L. J. 2018 | 1 | 1 | 1 | 1 | 1 | 1 | 0 | 0 | 0 | 0 | 0 | 6 |
| Mohammadifard, M. 2019 | 1 | 1 | 1 | 1 | 1 | 1 | 0 | 0 | 0 | 0 | 0 | 5 |
| Okushin, K. 2015 | 1 | 1 | 1 | 1 | 1 | 1 | 1 | 1 | 0 | 0 | 0 | 8 |
| Polyzos, S. A. 2013 | 1 | 1 | 1 | 1 | 1 | 1 | 0 | 0 | 0 | 0 | 0 | 5 |
| Yu, Y. Y. 2018 | 1 | 1 | 1 | 1 | 1 | 1 | 1 | 1 | 0 | 0 | 0 | 7 |
| Alvarez, C. S. 2020 | 1 | 1 | 1 | 1 | 1 | 1 | 1 | 1 | 0 | 0 | 0 | 8 |
| Abo-Amer, Y. E.2020 | 1 | 1 | 1 | 1 | 1 | 1 | 1 | 0 | 0 | 0 | 0 | 6 |
| Wang, J.W. 2021 | 1 | 1 | 1 | 1 | 0 | 1 | 1 | 0 | 1 | 1 | 1 | 9 |
| A= Define the source of information (survey, record review); B= List inclusion and exclusion criteria for exposed and unexposed subjects (cases and controls) or refe rto previous publications; C= Indicate time period used for identifying patients; D= Indicate whether or not subjects were consecutive if not population-based; E= Indicate if evaluators of subjective components of study were masked to other aspects of the status of the participants; F= Describe any assessments undertaken for quality assurance purposes (e.g., test/retest of primary outcome measurements); G= Explain any patient exclusions from analysis; H= Describe how confounding was assessed and low controlled; I= If applicable, explain how missing data were handled in the analysis; J= Summarize patient response rates and completeness of data collector; K= Clarify what follow-up, if any, was expected and the percentage of patients for which incomplete data or follow-up was obtained. | | | | | | | | | | | | |

**Table S4 The covariates in included studies**

| **Study** | **Effective size** | **Country of** **participants** | **The covariates adjustment** |
| --- | --- | --- | --- |
| Abdel-Razik, A. 2018 | HR | Egypt | Sex, age, BMI, smoking, crowding index, education level, and regular exercise, CRP, IL-6 and TNF-α, HOMA-IR, FPG, total cholesterol, HDL-C, LDL-C, triglycerides, and uric acid, leptin, adiponectin, and LAR |
| Baeg, M. K. 2016 | OR | South Korea | Sex, body mass index, diabetes, insulin, or metabolic syndrome components |
| Cai, O. 2018 | OR | China | Sex, BMI, TG, HDL-C, FPG |
| Chen, C. X. 2017 | OR | China | Sex, age, uric acid, AST, ALT, γ-glutamyltransferase, triglyceride, body mass index, waist circumference, and serum HbA1C |
| Doulberis, M. 2020 | OR | Switzerland | NA |
| Fan, N. 2018 | OR | China | BMI, SBP, and DBP |
| Jiang, T. 2019 | OR | China | Sex, age, education level, smoking, hypertension, diabetes, dyslipidemia, BMI, ALT, AST, AKP, TBIL, UA, and UREA |
| Kang, S. J. 2018 | OR | USA | Age, sex, race-ethnicity, income, diabetes, hypertension, smoking status, waist circumference, alcohol consumption, caffeine consumption, total cholesterol, high-density lipoprotein-cholesterol |
| Kim, T. J. 2017 | HR | South Korea | Age, sex, body mass index, year of screening exam, smoking status, alcohol intake, regular exercise, education level, fasting blood glucose, triglycerides, LDL-C, and HDL-C |
| Kumar, R. 2017 | OR | India | NA |
| Lecube, A. 2016 | OR | Spain | NA |
| Lu, L. J. 2018 | OR | China | NA |
| Mohammadifard, M. 2019 | OR | Iran | NA |
| Okushin, K. 2015 | OR | Japan | NA |
| Polyzos, S. A. 2013 | OR | Greece | NA |
| Shen, Z. 2013 | OR | China | NA |
| Sumida, Y. 2015 | OR | Japan | BMI body mass index, IGT impaired glucose tolerance |
| Tang, D. M. 2019 | OR | USA | Age, sex, and statin use |
| Xu, M. Y. 2020 | OR | China | Age, gender, underlying diseases, and MS |
| Yu, Y. Y. 2018 | OR | China | NA |
| Zhang C. 2016 | OR | China | Gender and age disease |
| Alvarez, C. S. 2020 | OR | Guatemala | Age, sex, education, residence, smoking, alcohol intake, and, for all conditions excluding obesity and central obesity, BMI |
| Abo-Amer, Y. E. 2020 | OR | Egypt | NA |
| Wang, J.W. 2021 | OR | China | PGI, PGII, PGI/PGII, pro-PG |
| Yan, P. 2021 | OR | China | Age, carotid plaque status, alanine aminotransferase, aspartate aminotransferase, uric acid levels, fasting plasma glucose, total cholesterol, triglyceride, systolic blood pressure, diastolic blood pressure, low-density lipoprotein cholesterol, and body mass index |

NA: Not Applicable

**Figure S1 The sensitivity analysis of the meta-analysis in association between HP infection and NAFLD with HR**





**Figure S2 The sensitivity analysis of the meta-analysis in association between HP infection and NAFLD with OR for Asian**





**Figure S3 The sensitivity analysis of the meta-analysis in association between HP infection and NAFLD with OR for non-Asian**





**Figure S4 The publication bias**


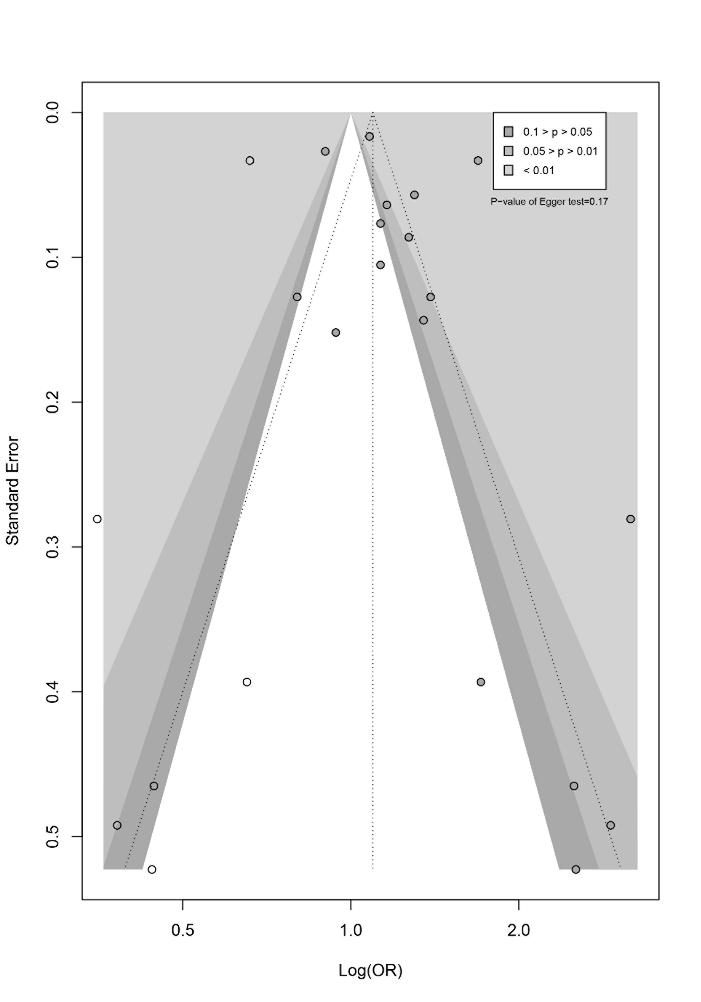

Supplement: Supplementary file 1 [file Data_Sheet_1.docx]
